# Pierre Fauchard (1678-1761): Pioneering Dental Surgeon of the Enlightenment Age

**DOI:** 10.7759/cureus.69563

**Published:** 2024-09-16

**Authors:** Umut Aksoy, Seçil Aksoy, Dilan Kırmızı, Kaan Orhan

**Affiliations:** 1 Endodontics, Faculty of Dentistry, Near East University, Nicosia, TUR; 2 Dentomaxillofacial Radiology, Faculty of Dentistry, Near East University, Nicosia, TUR; 3 Dentomaxillofacial Radiology, Faculty of Dentistry, Ankara University, Ankara, TUR

**Keywords:** biography, dentistry, historical vignette, history of dentistry, surgeon dentist

## Abstract

Pierre Fauchard, widely referred to as the "Father of Modern Dentistry," fundamentally transformed the field with his seminal 1728 publication, *Le Chirurgien Dentiste, ou Traité des Dents.* Born circa 1677 in Brittany, France, Fauchard's early exposure to severe dental conditions during his naval service catalyzed his pursuit of advancements in dental science. Upon transitioning from naval service to establish a practice in Angers, and subsequently gaining acclaim in Paris, Fauchard systematically documented and organized dental practices, encompassing oral surgery, orthodontics, periodontics, and prosthodontics, thereby laying the foundational framework for contemporary dental practices.

Fauchard's innovations included the use of materials such as lead, tin, and gold for dental fillings and the introduction of early orthodontic techniques, notably the *Bandeau.* His treatise also emphasized the importance of preventive care and oral hygiene, which provided a basis for modern dental hygiene protocols. Additionally, Fauchard's critical evaluation of fraudulent practices and his inclusion of numerous clinical case studies in his treatise bridged theoretical knowledge with practical application, significantly impacting dental education and professional standards.

Fauchard's influence extends beyond national boundaries, profoundly shaping global dental practices and educational frameworks. The Pierre Fauchard Academy, established in 1936, continues to uphold his principles, underscoring the enduring relevance of his contributions. Fauchard’s work remains a cornerstone of modern dentistry, reflecting his profound and lasting impact on the discipline.

## Introduction and background

Early life and education

Pierre Fauchard, often heralded as the "Father of Modern Dentistry," was born around 1677 or 1678, in Brittany, France [1]. The exact details of his early life remain obscure, largely because Fauchard did not document much about his personal background or family origins. Nevertheless, it is widely believed that his family name, Fauchard, traces its roots back to Brittany, and may have been derived from a medieval weapon known as a fauchard, a long wooden pole with a curved blade [2].

At the rather young age of 15, circa 1693, Fauchard embarked on a career that would later shape his influence in dentistry. He enlisted in the French Royal Navy as an apprentice surgeon during the era of Louis XIV [3]. Part of the reason for this decision was financial necessity; education in surgery at this time was expensive and not readily available [4]. He was in the navy, and onboard, he was a mentee to Alexandre Poteleret, who was the Surgeon-Major. Poteleret was very interested in oral diseases and, through his knowledge and training, brought oral health and dentistry to the attention of Fauchard. With this old sea dog, Fauchard recorded many oral diseases common among sailors such as scurvy [3,4]. It is from this practical experience that he identified many innovations in dentistry.

Fauchard's naval service lasted approximately three years, during which he honed his skills and knowledge through rigorous practical training and extensive reading. His early exposure to the severe dental conditions faced by sailors due to poor nutrition and hygiene instilled in him a desire to pursue advancements in dental care [5].

After leaving the navy in 1696, Fauchard set up his dental practice in the large university town of Angers [5]. He is thought to have been the first to use the term "chirurgien-dentiste," distinguishing his practice from the much cruder role of the "dentateurs" or "denture makers" of the time [3]. His innovative approach and determination to improve dental care brought him a reputation for excellence. He then spent the next 20 years or so in practice and in improving his trade in regions such as Nantes, Tours, and Rennes [1,5].

Around 1718, Fauchard was comfortably settled and recognized as one of the best dental surgeons in Paris. He was characterized by having solid practical skills and possessing novel methods to treat various oral illnesses. His colleagues, among even general surgeons of the time, often invited him for consultations with patients afflicted with diseases related to teeth. This was the point in time when Fauchard began to assume leadership status in the dental community [6].

With the publication of a major work, "Le Chirurgien Dentiste, ou Traité des Dents" (The Surgical Dentist, or Treatise on the Teeth) [7], his leadership stature was firmly established. The book was the first to systematically document dental practices, diseases, and treatments, transforming dentistry from a trade practiced by barbers and blacksmiths into a scientific profession [4].

## Review

“Le Chirurgien Dentiste, ou Traité des Dents”: work and innovations

**Figure 1:**
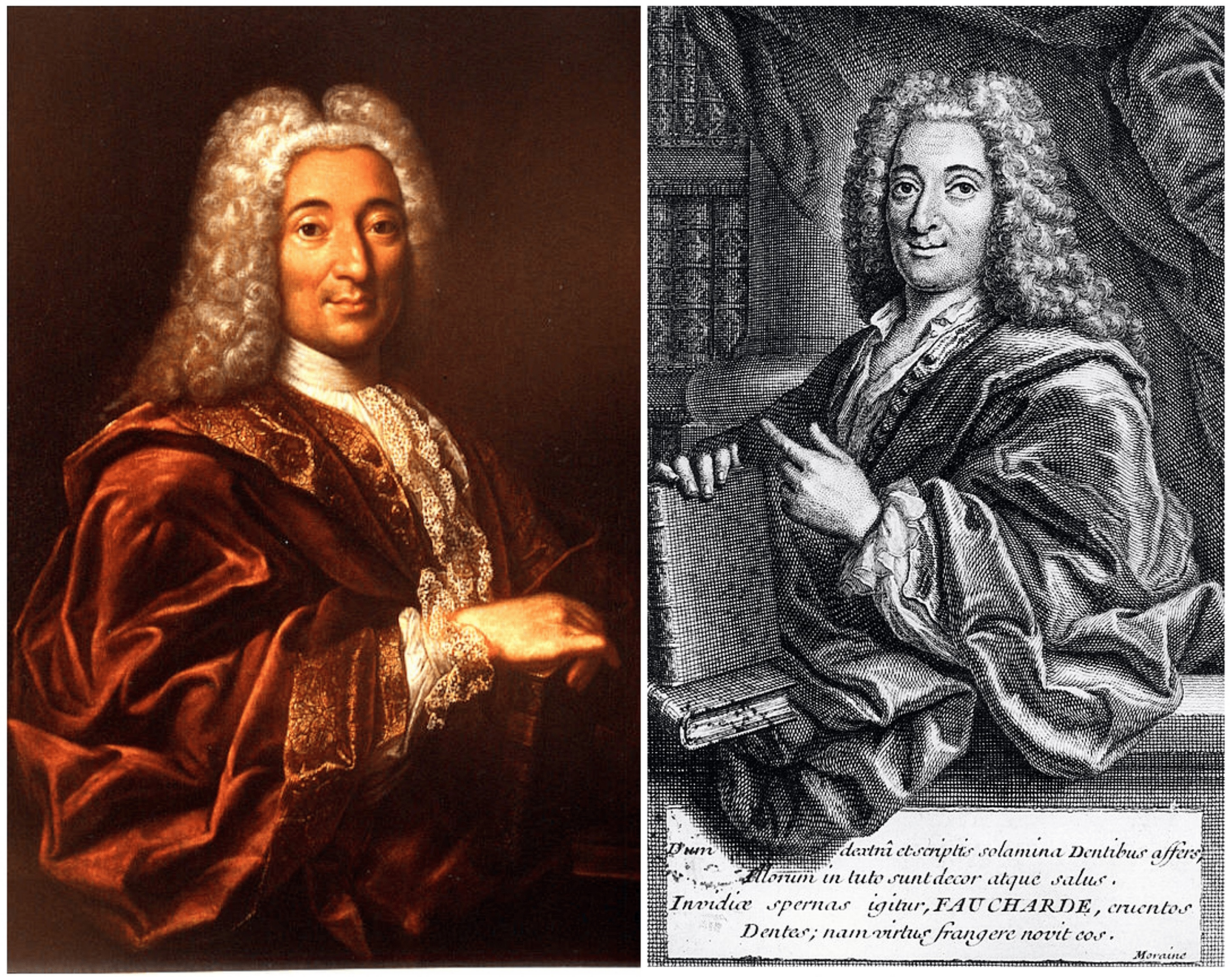
Pierre Fauchard (1678-1761), the Father of Modern Dentistry, and the first page of "Le Chirurgien Dentiste," 1st ed., 1728. The Latin inscription reads: “Dum dextra et scriptis solamina dentibus affers illorum in tuto sunt decor atque salus. Invidiae spernas igitur, FAUCHARDE, cruentos dentes; nam virtus frangere novit eos.” Translated into English: “While you bring comfort to teeth with your right hand and writings, their beauty and health are secure. Therefore, FAUCHARD, scorn the bloody teeth of envy; for virtue knows how to break them.” (Images from Wikimedia Commons [8], the free media repository)

Pierre Fauchard, wrote his magnificent book, "Le Chirurgien Dentiste, ou Traité des Dents" (The Surgeon Dentist, or Treatise on the Teeth), at 45 years of age, in 1723 (Figure 1).

The book, after careful examination, was published in 1728 and appeared first in French and later in German in 1773. Despite its early translation into German, the first English translation was not published until 1946. This over-800-page volume, a treatise in two parts consisting of 64 chapters, dominated dentistry as the "bible" of the field for nearly 100 years, providing dentists the world over with a common language. Fauchard's book contained 42 plates, providing illustrations of many instruments and appliances that he used. In 1746, the date of the second edition of Fauchard's work, a complete revision of the book, was undertaken and the style vastly improved through the medium of notes to clarify many of his previous statements, and much new material was added, while at the same time unnecessary repetitions were omitted, thus increasing the text portion by 61 pages. It is in this edition that he gave his famous clinical description of pyorrhea alveolaris, though the disease itself, he states, was known for hundreds of years. His book provides an exhaustive description of 103 diseases of the teeth and oral cavity, an impressive and extensive catalog for any textbook of that era [2].

The treatise covered an astonishing breadth of knowledge in dentistry, including oral surgery, orthodontics, periodontics, and prosthodontics, as well as basic sciences such as anatomy, pathology, and pharmacology. His advancements turned dental practice from routine procedures into a more scientific and systematic approach, laying the foundation for modern dentistry [2,3].

Fauchard's treatise was the first of its kind, and during its time, it was the only treatise on dental science and practice that was comprehensive and systematic. In that text, he elaborated in detail the anatomy of the mouth, the dental diseases, and their cures and thus set up a scientific and organized method of dentistry, which was non-existent earlier. Fauchard organized the hitherto scattered knowledge of dental surgery into a practical guide for the operator. It ranged from the anatomy of teeth and oral cavity, pathologies on the mouth, and operative and prosthetic techniques, thus making it a reference indispensable to dental practitioners of his time and beyond [1,2].

The range of Fauchard's work is so immense that it can't be overemphasized. He discussed many dental instruments and techniques in his book, which effectively defined and hence standardized the tools and procedures that were used in dental practice. As in dentistry, this certainly was an improvement from earlier methodologies that tended to be inconsistent and, at times, injurious. In this manner, Fauchard set a new level of standard for education and practice in dentistry by having such techniques documented and systematized, ensuring that later generations of dentists would be standing on solid ground [1,2].

Innovations in dental techniques

Fauchard implemented several novel methods that have since become the basis of modern-day dental practice. One among them was his contribution to the field of dental prosthetics. He delineated processes in which artificial teeth and prosthetic equipment could be fabricated to add both form and function to dental restorations. He took materials like ivory and human teeth to make dentures, fixing them with gold wire and waxed thread. This was far better than the primitive mechanisms utilized earlier, constituting the very foundation for contemporary prosthetic dentistry [4].

Aside from prosthetics, Pierre Fauchard pioneered the invention of cavity fillings and methods for treating tooth decay. In the eighteenth century, he recommended using metals such as lead, tin, and gold for dental fillings, with a particular preference for tin. He demonstrated that thoroughly removing all traces of caries before filling the tooth would strengthen it, a principle that became a cornerstone of modern dental practice. To treat cavities, Fauchard employed files, an Archimedes-type drill, and sharp instruments to enlarge the cavity and remove decay. He would then cauterize the cavity with a hot instrument before filling it [9]. Fauchard was among the first to dismiss the idea that worms in teeth caused decay, instead attributing dental caries to acids derived from sugar. He carefully removed infected tissue and sealed the pulp with lead foil months following the treatment of root canals exhibiting purulent inflammation. Additionally, Fauchard described the use of a dental light over the dental chair and made clear drawings of a functional dental drill. His theory of acidic sugars creating decay, and his innovative concepts for procedures and materials made a foundation that moved dentistry beyond its origins and into what is practiced today [7].

Fauchard's treatise contained early theories on orthodontics, and he was the first to give a scientific account of such practices [10]. He described methods used to correct anomalies in dentition and irregularities such as devices used for straightening teeth. An example of this is the "Bandeau," a horseshoe-shaped metal orthodontic strip constructed for the expansion of the arch. The arch was ligated to the teeth by waxed silk ligatures, which exerted pressure on the teeth and expanded the arch progressively with a lead of one-eighth (1/8th) of an inch. This early orthodontic method paved the way for new improvements in contemporary orthodontics and became the foundation for Angle's E-Arch [11]. In this regard, Fauchard's work is quite clear in saying that there needs to be the proper alignment of teeth: deviation from such an alignment may bring about negative effects on the general status of the oral cavity.

Also crucial for Fauchard's work was the description of surgical procedures in the treatment of dental conditions, which included the extraction of teeth. His comprehensive dental treatise gave an all-encompassing view of the anatomy of both teeth and gums, thereby creating the anatomy of extraction tools that are specifically designed to fit various shapes and functions of different sets of teeth. It was this innovation that heralded the era of elevators and forceps designed for dental extractions, and therefore a more controlled and effective extraction process ensued. He stressed to perform exacting and precise dental surgery procedures, and his standards became a reducing principle for complications in dentistry practice. He also advocated replanting avulsed teeth due to the fact that they were potentially long-term restorations, and consequently, he gained the status of the first dental traumatologist [6]. In addition, Fauchard introduced a revolutionary change regarding patient care. He proposed that patients should be treated in an armchair, unlike the traditional approach where patients used to sit on the floor. His methodical approach to dental surgery, grounded in extensive clinical experience, was meticulously documented in his book, setting a new benchmark for dental practice.

Dental instruments and tools

As dentistry entered a restorative phase at the beginning of the eighteenth century, instruments to remove caries gained importance and variety. In his seminal 1728 book, Pierre Fauchard depicted four different scrapers and excavators designed for this purpose [12]. He defined many tools and described some that he made himself. He developed special forceps and drills that became the pioneers of contemporary dental devices. Fauchard outlined their purposes and the modifications made from the initial models. For instance, he created an improved dental drill whose rotary movement was powered by catgut twisted around a cylinder or a jeweler’s bowstring [7,13]. Fauchard introduced specialized forceps and elevators for tooth extraction, facilitating safer and more effective removal of teeth while reducing patient discomfort. These instruments significantly improved the efficiency of dental treatments and set a new benchmark for the development of dental tools [7].

Critique of charlatan practices

Fauchard was also highly critical of the fraudulent practices of contemporary dental charlatans. He sought to elevate the standards of dental care by exposing these deceitful practices and advocating for scientific rigor and ethical behavior in the practice of dentistry [3]. His critique of the neglect shown by surgeons toward dental care highlighted the need for a specialized and professional approach to dentistry. Fauchard lamented in the following words: "*The most famous surgeons having abandoned this part of the art have caused by this negligence, the rise of people who without theory or experience, have degraded it, and practiced haphazard, without principles or method*” [7].

Preventive dentistry and oral hygiene

Fauchard was one of the first to realize the significance of preventive dentistry and oral hygiene. He was an advocate for cleaning teeth at regular intervals to avoid decay and gum diseases. Indeed, the treatise went further by expounding that oral hygiene should be maintained through washing the mouth every morning with tepid water and rubbing the teeth with a fine sponge wet in water or a mixture of water and aqua vitae (a kind of alcohol). He also came up with a much milder recipe for dentifrice, containing coral, dragon's blood, burnt honey, and cuttlefish bone - believed to clean the teeth without harming them [14].

Fauchard's vision of preventive dentistry was relatively revolutionary. He recognized that good oral hygiene would save one from dental disease and advocated visiting the dentist regularly at an interval of four to six months [3]. This approach to prevention laid the foundation for modern dental hygiene and gave a strong emphasis to the need for professional maintenance consistently in preventing dental disease.

Case histories and clinical practice

In his book, Fauchard has included numerous case histories that expressed much about his clinical work [15]. These cases presented clear examples of his diagnostic abilities, procedures for treatment, and the diversity in the number and variety of dental complaints he handled [7]. At that time, such practical examples in medical literature were groundbreaking to bridge theoretical knowledge with practical application.

Educational contributions

Fauchard's treatise became a source of education for generations of future dentists. It formed the basics for establishing dental schools and professional training courses. By recording his knowledge and clinical experience, Fauchard created a manual that aspiring dentists could use to learn dental principles and techniques. His clear and practical style of teaching was emphasized in the system of education, combined with extensive study and high manual dexterity, which set the standards for modern dental education.

Impact and legacy

The influence of Fauchard extended beyond France, becoming an international foundation for dental practice. The basic principles were carried from France to the United States by James Gardette, a French naval surgeon, who studied under esteemed Parisian dentists influenced by Fauchard and became the first medically trained dentist to regularly treat American military personnel [5].

The Pierre Fauchard Academy, founded in 1936 by Dr. Elmer S. Best, a Minnesota dentist, continues to uphold Fauchard's principles. Dr. Best, troubled by the proprietary nature of many dental publications, sought to help the profession gain control of its own literature and ensure its independence from commercial interests. His dedication to learning and advancing professional practices became the underpinning of the Academy. It's been able to attract the finest dental researchers and educators in this country, and it fosters dental science with good ethics among its practitioners [16]. Fauchard's contributions laid down not only the philosophy in which modern dentistry is steeped but also served as an impetus for a tradition that would propagate scientific research and cooperation among dental practitioners. His work was such a monumental influence in the development of dentistry, not just in North America but also around the world, that professional standards were set and a professional identity for dentists firmly created.

## Conclusions

The work "Le Chirurgien Dentiste, ou Traité des Dents," by Pierre Fauchard, laid down the guidelines for the fundamental landmark of the dentistry field. His methodical treatment of dental science brought a revolution and improved techniques for better preventive care in the field. Many of his principles now lie as the cornerstone upon which modern dental practice is based. His legacy is reflected in today's dentists adopting his methods and professional identity. The works of Fauchard went a long way to create a sustained influence in the field of dental health care because they made dentistry to be of the rank of a known and scientific discipline, such that his name remains synonymous with the advancement of dental science.
